# HIV Infection of Hepatocytes Results in a Modest Increase in Hepatitis C Virus Expression *In Vitro*

**DOI:** 10.1371/journal.pone.0083728

**Published:** 2014-02-28

**Authors:** Ling Kong, Jeffrey A. Welge, Eleanor A. Powell, Jason T. Blackard

**Affiliations:** 1 Division of Digestive Diseases, Department of Internal Medicine, University of Cincinnati College of Medicine, Cincinnati, Ohio, United States of America; 2 Departments of Psychiatry and Environmental Health, University of Cincinnati College of Medicine, Cincinnati, Ohio, United States of America; Saint Louis University, United States of America

## Abstract

Previous studies demonstrate that soluble HIV proteins impact both hepatocyte function and HCV replication *in vitro*. It has also been reported that HIV can productively infect hepatocytes. We therefore investigated the impact of HIV infection of hepatocytes on HCV expression. The Huh7.5_JFH1_ cell line that constitutively expresses infectious HCV was infected with the lab-adapted strains HIV_NL4-3_ or HIV_YK-JRCSF_. HCV expression was quantified via HCV core antigen ELISA, Western blot, and strand-specific real-time PCR for positive-sense and negative-sense HCV RNA. After HIV_NL4-3_ infection of Huh7.5_JFH1_ cells, positive-sense and negative-sense HCV RNA levels were elevated compared to HIV uninfected cells. Increased HCV RNA synthesis was also observed after infection of Huh7.5_JFH1_ cells with HIV_YK-JRCSF_. HIV-induced HCV core production was decreased in the presence of the anti-HIV drugs AZT, T20, and raltegravir, although these medications had a minimal effect on HCV expression in the absence of HIV. HCV core, NS3, and NS5A protein expression were increased after HIV infection of Huh7.5_JFH1_ cells. Chemically inactivated HIV had a minimal effect on HCV expression in Huh7.5_JFH1_ cells suggesting that ongoing viral replication was critical. These data demonstrate that HIV induces HCV RNA synthesis and protein production *in vitro* and complement previous *in vivo* reports that HCV RNA levels are elevated in individuals with HIV/HCV co-infection compared to those with HCV mono-infection. These findings suggest that HIV suppression may be a critical factor in controlling liver disease, particularly if the underlying liver disease is not treated.

## Introduction

Since the introduction of highly active antiretroviral therapy (HAART), liver disease – frequently caused by hepatitis C virus (HCV) co-infection – has surpassed AIDS-defining illnesses as a major cause of morbidity and mortality in HIV-positive persons [1], [2]. Multiple clinical studies have clearly demonstrated that HIV co-infection results in increased HCV RNA levels, progressive liver disease, and decreased HCV treatment response rates (reviewed in [3], [4]). HIV infection is associated with a number of hepatic and biliary tract disorders, hepatomegaly, hepatic steatosis, and elevated serum liver enzymes [5]–[8]. HIV-positive patients with no evidence of viral hepatitis co-infection also exhibit mild-to-moderate increases in liver enzyme levels [9]–[11]. Several groups, including our own, have also reported that plasma HIV RNA levels were associated with liver disease during HIV mono-infection, suggesting a direct involvement of HIV in liver disease [12]–[15].

The influence of HIV on HCV disease is not solely immune mediated, as HCV RNA levels are strongly associated with HIV RNA levels [16]. Thus, virus-virus interactions are likely. Such interactions may be mediated via 1) secreted HIV proteins that interact with HCV-infected hepatocytes, and/or 2) direct HIV infection of HCV-infected hepatocytes. However, until recently, *in vitro* systems to study the complete HCV life cycle were unavailable; therefore, our ability to explore HIV/HCV interactions were also limited. Recently, the advent of infectious HCV cell culture systems has spawned renewed interest in exploring the cellular pathways contributing to HIV/HCV pathogenesis. Moreover, the availability of distinct classes of anti-HIV agents that target viral entry, reverse transcription, integration, or infectious virion production allows for more detailed mechanistic studies of HIV-mediated HCV replication.

Several lines of evidence highlight the potential for HIV to interact directly with multiple liver cell populations (reviewed in [17], [18]). For example, HIV proviral DNA was detected in liver biopsies from patients with AIDS [19], while HIV proteins and viral RNA have been detected in hepatocytes, Kupffer cells, inflammatory mononuclear cells, and sinusoidal cells using liver samples from HIV-infected patients [19], [20]. Similarly, intracellular expression of HIV proteins has been reported in Kupffer cells, endothelial cells, and hepatocytes [21]–[23]. Once considered controversial because of a lack of data, several recent reports have convincingly demonstrated productive, low-level HIV infection of hepatocytes. Xiao *et al.* isolated an HIV strain from a patient with advanced disease that was able to infect hepatocytes [24]. Primary human hepatocytes were also susceptible to HIV infection. Fromentin *et al.* demonstrated that a hepatocyte-derived cell line binds to and internalizes HIV particles [25]. Furthermore, HIV infection of CD4^+^ T cells was enhanced after interactions with virus-loaded hepatocytes compared to cell-free virus. Iser *et al.* observed increased HIV reverse transcriptase activity following infection of hepatocyte cell lines with X4 or R5 HIV [26]. We recently demonstrated integrated HIV DNA in the Huh7.5 and Huh7.5_JFH1_ cell lines and primary hepatocytes that was inhibited by raltegravir in a dose-dependent manner [27]. HIV p24 protein was also detected in cell culture supernatants and was inhibited by AZT, although levels were modest compared to those in a lymphocyte cell line. The detection of HIV proteins and nucleic acids in hepatocytes is particularly interesting given that hepatocytes are the major site of HBV/HCV replication. Given these findings, we investigated whether HIV could regulate HCV expression in hepatocytes *in vitro*.

## Materials and Methods

### Cell lines and reagents

The Huh7.5_JFH1_ cell line – which produces infectious HCV genotype 2a virions – was provided by Dr. Guangxiang Luo [28] and maintained in DMEM high glucose medium supplemented with 10% FBS, penicillin (100 U/mL), and streptomycin (100 mg/mL). The following reagents were obtained through the AIDS Research and Reference Reagent Program, Division of AIDS, NIAID, NIH: AZT, T20 from Roche, TZM-bl cells [29] from Drs. John Kappes and Xiaoyun Wu and Tranzyme Inc., plasmids pNL4-3 [30] from Dr. Malcolm Martin and pYK-JRCSF [31] from Drs. Irvin Chen and Yoshio Koyanagi, and raltegravir from Merck & Company, Inc. 293T cells were obtained from ATCC.

### Virus preparation

HIV_NL4-3_ (X4-tropic) and HIV_YK-JRCSF_ (R5-tropic) were prepared by transfection of 1×10^6^ 293T cells per well in a 24-well plate with 1 ug of the appropriate full-length infectious HIV plasmid using the FuGene6 transfection reagent (Roche). Transfected cells were incubated at 37°C for an additional 48–72 hours. Virus-containing supernatants were passed through a 0.20 um filter to remove cellular debris and precipitated in polyethylene glycol at 4°C. Precipitated virus was then centrifuged at 14,000 g for 20 minutes, resuspended in PBS, and frozen at −80°C until use. The level of p24 protein in cell culture supernatants was determined by p24 ELISA (Perkin-Elmer; Boston, MA; lower limit of detection = 4.3 pg/mL) or by titering on TZM-bl cells.

### AT-2 treatment of HIV

For preparation of non-infectious HIV with functionally intact envelope glycoproteins, HIV_NL4-3_ and HIV_YK-JRCSF_ were first prepared by transfection of 293T cells as described above. After filter sterilization, virus was inactivated by adding 250 uM of aldrithiol-2 (AT-2; Sigma-Aldrich) to the filtered supernatants as described elsewhere [32]. AT-2-treated viruses were then incubated with the TZM indicator cell line to confirm that they were not infectious (data not shown). HIV-1 p24 ELISA was measured in treated virus preparations.

### HIV infections

1×10^5^ Huh7.5_JFH1_ cells were seeded per well of a 96-well, collagen-coated plate and incubated at 37°C in 5% CO_2_ with infectious HIV at a multiplicity of infection [MOI] of 1.0 in a volume of ∼100 uL. After 2 hours, unbound viruses were removed by washing cells five times with phosphate buffered saline, and fresh medium was added. Cells and cell culture supernatants were removed at various time points post-infection to measure HCV RNA or protein as described below. For experiments with antiretroviral agents, cells were incubated with 100 uM of AZT, 1 ug/mL of T20, or 1–100 nM raltegravir for one hour before and during HIV infection. For experiments with AT-2-treated HIV, Huh7.5_JFH1_ cells were incubated as described above in the presence of 0–200 ng of HIV as measured by HIV p24 ELISA.

### Quantification of HCV RNA and protein expression

Real-time PCR conditions to quantify strand-specific HCV RNA levels have been described in detail previously [33]. Briefly, RNA was extracted from 140 uL of culture supernatant using the QIAamp Viral RNA Kit (Qiagen; Valencia, CA), and eluted in 60 uL of DEPC-treated dH_2_O. For cell lysates, HCV RNA values (diluted 1∶1000 for positive-sense RNA and 1∶100 for negative-sense RNA) were normalized to the copy number of a housekeeping gene, glyceraldehyde-3-phosphate dehydrogenase (GAPDH) and presented as fold change. For Western Blots, 100,000 cells were harvested at days 3, 5, 7, and 14 post-infection and lysed in 100 uL of buffer; 10 uL was loaded per well. HCV NS3 and NS5A proteins were detected using a mouse monoclonal antibody (Ab) against HCV NS3 (ab18671; Abcam; Cambridge, MA) or a rabbit polyclonal Ab against HCV NS5A (ab2594; Abcam) as the primary Ab and a rabbit polyclonal IgG Ab as the secondary Ab (ab6921; Abcam). As an additional loading control, glyceraldehyde 3-phosphate dehydrogenase (GAPDH) was detected using a rabbit polyclonal Ab from Santa Cruz Biotechnology (SC-25778; Santa Cruz, CA). HCV core antigen was quantified in cell culture supernatants by the QuikTiter HCV Core Antigen ELISA Kit (Cell Biolabs, Inc.; San Diego, CA) or the Ortho HCV Antigen ELISA Test Kit (Ortho Clinical Diagnostics; Tokyo, Japan).

### Statistical analyses

Graphical inspection and basic descriptive statistics were used to check for potential outlying observations or violations of assumptions for parametric statistical models. Analysis of variance (ANOVA) was used to assess variation among experimental conditions. The choice between heterogeneous or homogeneous residual variance among experimental conditions was made in each experiment on the basis of the Akaike Information Criterion. In order to balance Type I errors against the relatively low power afforded by the small sample size, P values≤0.10 are reported. SAS PROC MIXED version 9.2 was used for all inferential analyses.

## Results

### HIV infection of Huh7.5_JFH1_ cells increases HCV RNA synthesis

We previously reported that integrated HIV DNA was detected in Huh7.5 and Huh7.5_JFH1_ cells, as well as in primary hepatocytes, after *in vitro* infection [27]. Several antiretroviral agents inhibited HIV infection of hepatocytes, although HIV levels were modest compared to those in a lymphocyte cell line. Culture supernatants from HIV-infected hepatocytes were capable of infecting a non-hepatic HIV indicator cell line suggesting production of infectious HIV from hepatocytes. In the current study, we examined the effects of HIV infection on HCV replication in Huh7.5_JFH1_ hepatocytes.

**Figure 1:**
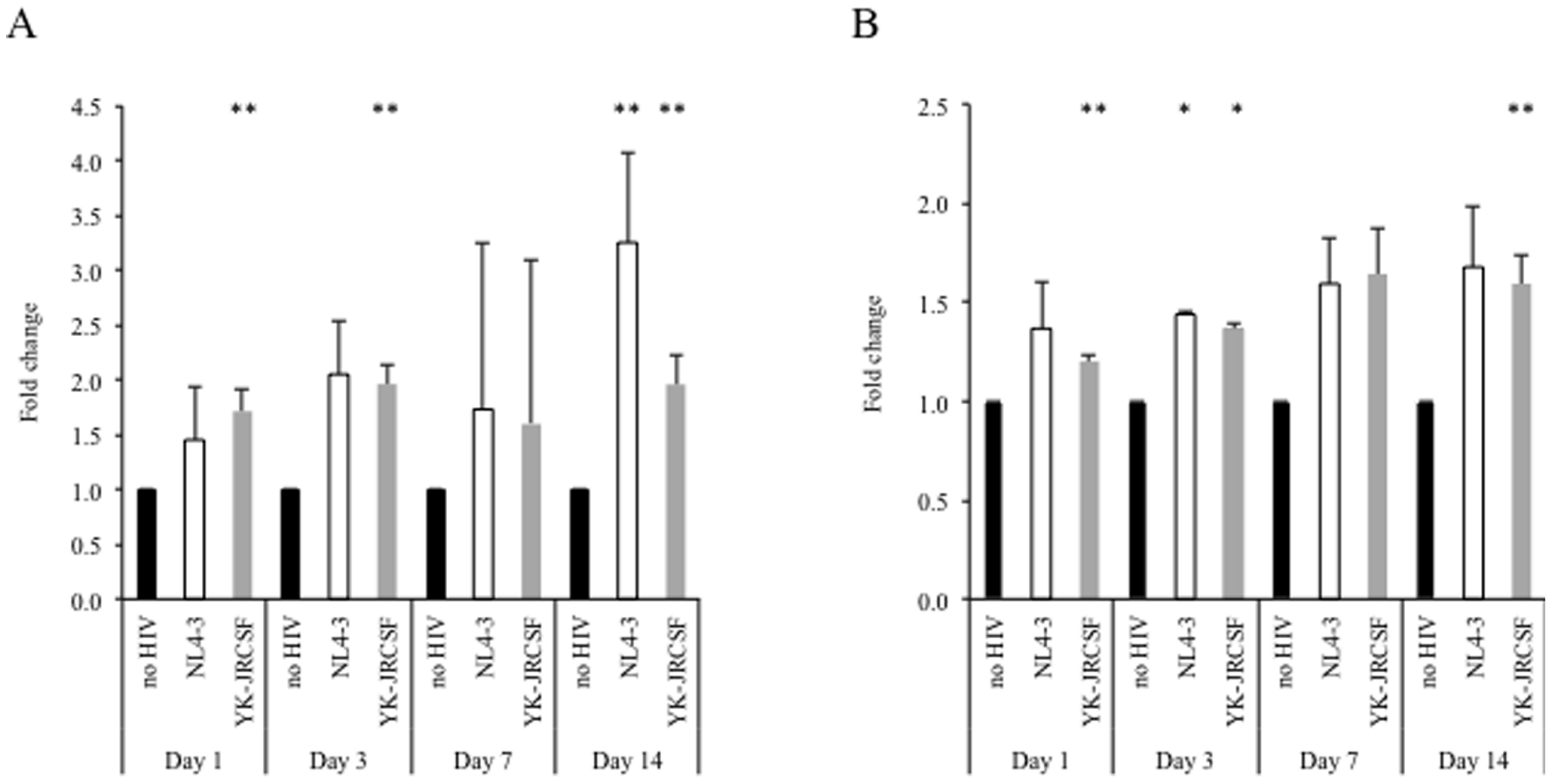
Infection of Huh7.5_JFH1_ cells with HIV results in increased HCV RNA synthesis. 100,000 Huh7.5_JFH1_ cells were incubated with DNase-treated HIV_NL4-3_ (white bars) or HIV_YK-JRCSF_ (grey bars) at an MOI = 1 for two hours. At days 1, 3, 7, and 14 post-infection, positive-sense HCV RNA (**A**) and negative-sense HCV RNA (**B**) were quantified in cell lysates, compared to the no HIV condition (black bars) by real-time, strand-specific PCR, and normalized to cellular GAPDH levels as described previously [33]. * p≤0.05; ** p≤0.10 compared to HIV-uninfected Huh7.5_JFH1_ cells at each time point.

HCV RNA was quantified at days 1, 3, 7, and 14 post-infection with HIV_NL4-3_ or HIV_YK-JRCSF_ by strand-specific real-time PCR. As shown in Figure 1A–B, both positive-sense and negative-sense HCV RNA were elevated in the presence of the HIV. Increased HCV RNA expression showed a trend towards statistical significance for positive-sense RNA in the presence of HIV_NL4-3_ at day 14 and in the presence of HIV_YK-JRCSF_ at days 1, 3, and 14. Similarly, increased negative-sense RNA was statistically significant in the presence of HIV_NL4-3_ at day 3 and in the presence of HIV_YK-JRCSF_ at day 3. As well, there was a trend towards statistical significance in the presence of HIV_YK-JRCSF_ at days 1 and 14.

**Figure 2:**
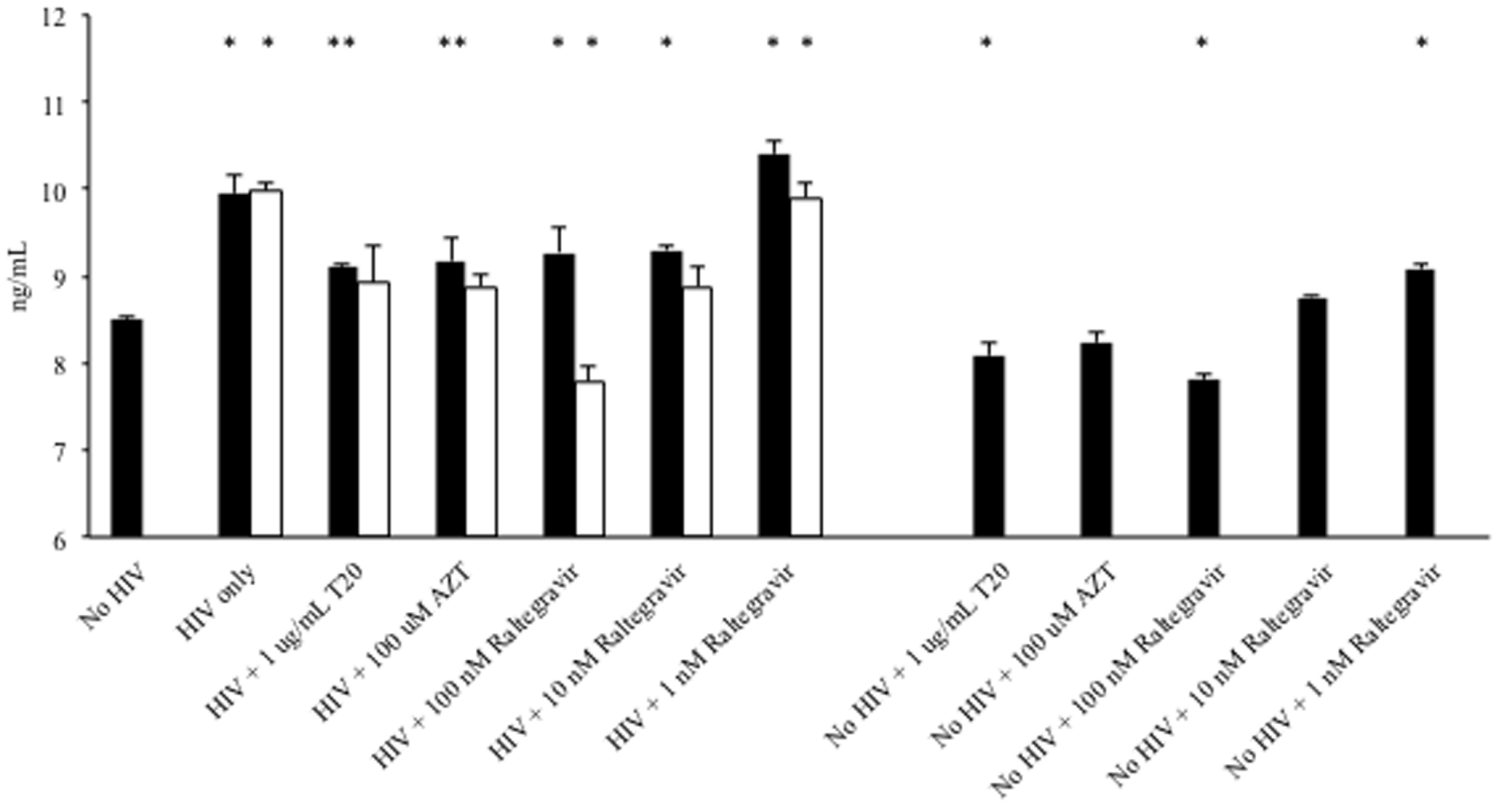
HIV infection of Huh7.5_JFH1_ cells is inhibited by anti-HIV medications. 100,000 Huh7.5_JFH1_ cells were incubated with DNase-treated HIV_NL4-3_ (black bars) or HIV_YK-JRCSF_ (white bars) an MOI = 1 for two hours. Cells were pre-treated with AZT (100 uM), T20 (1 ug/mL), or raltegravir (1–100 nM) for one hour and then during HIV infection and incubation. At day 3 post-infection, HCV core antigen was quantified in cell lysates. * p≤0.05; ** p≤0.10 compared to HIV-uninfected Huh7.5_JFH1_ cells under each treatment condition.

Pre-treatment of cells with the reverse transcriptase inhibitor AZT (100 uM), the entry inhibitor T20 (1 ug/mL), or the integrase inhibitor raltegravir (1–100 nM) resulted in decreased HCV core antigen production in the presence of HIV_Nl4-3_ and/or HIV_YK-JRCSF_ (Figure 2). In the absence of HIV infection, a modest but statistically significant impact of certain HIV medications on HCV expression was observed.

### HIV infection of Huh7.5_JFH1_ cells increases HCV protein expression

**Figure 3:**
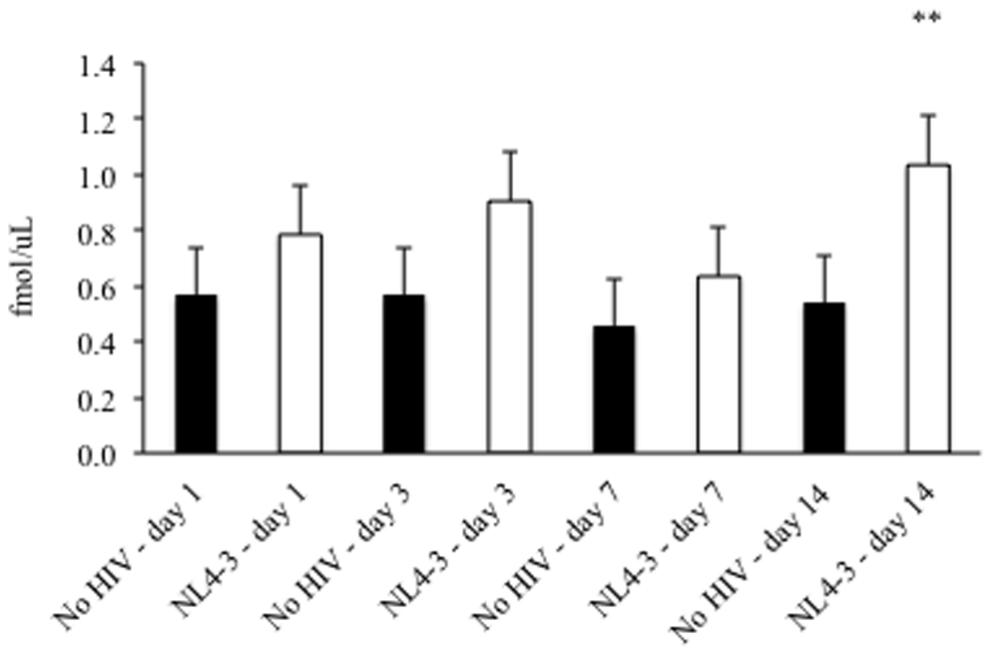
Infection of Huh7.5_JFH1_ cells with HIV results in increased HCV core protein production. 100,000 Huh7.5_JFH1_ cells were incubated with DNase-treated HIV_NL4-3_ at an MOI = 1 for two hours. At days 1, 3, 7, and 14 post-infection, HCV core antigen was quantified in cell lysates in the absence (black bars) or presence (white bars) of HIV. * p≤0.05; ** p≤0.10 compared to HIV-uninfected Huh7.5_JFH1_ cells at each time point.

**Figure 4:**
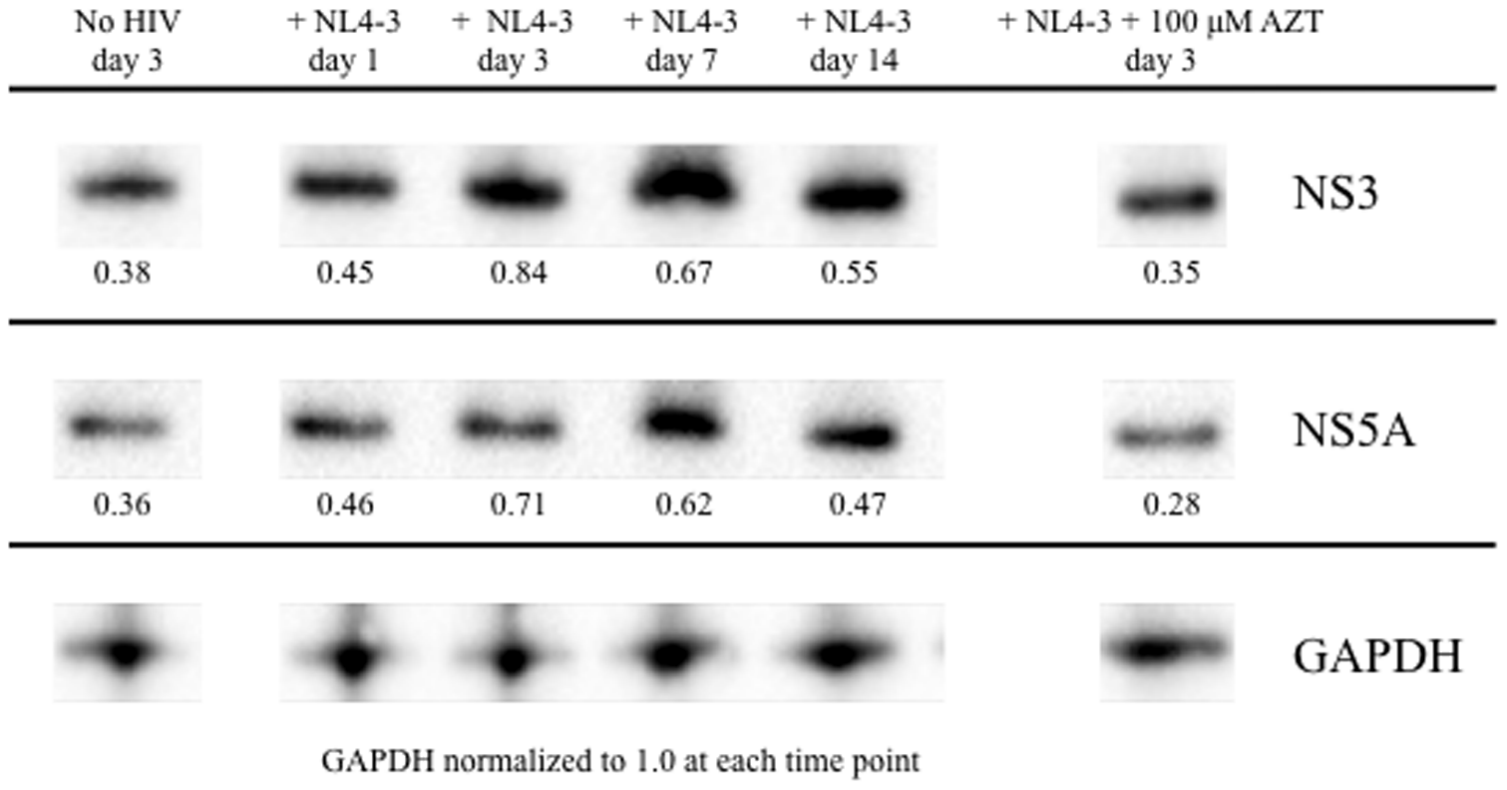
Infection of Huh7.5_JFH1_ cells with HIV results in increased HCV NS3 and NS5A protein production. 100,000 Huh7.5_JFH1_ cells were incubated with DNase-treated HIV_NL4-3_ at an MOI = 1 for two hours. Cell lysates were harvested at days 1, 3, 7, and 14 post-infection for the detection of HCV NS3 and NS5A proteins by Western Blot. Equivalent total cell protein concentrations were loaded, and all data were normalized to cellular GAPDH levels at each time point. The uninfected (no HIV) condition, as well as infection with HIV_NL4-3_ in the presence of 100 uM AZT, are shown as additional controls.

At days 1, 3, 7, and 14 post-infection with infection with HIV_NL4-3_, HCV core antigen expression was quantified in cell lysates and shown to be elevated in the presence of HIV (Figure 3). Additionally, increased HCV NS3 and NS5A protein expression were observed via Western blot in Huh7.5_JFH1_ cells infected with HIV_NL4-3_ at days 1, 3, 7, and 14 post-infection compared to uninfected control levels after normalization to GAPDH protein levels (Figure 4). Relative HCV NS3 and NS5A protein levels were significantly reduced at day 3 when HIV_NL4-3_ infection was performed in the presence of the antiretroviral drug AZT (0.84 versus 0.35 and 0.71 versus 0.28, respectively).

### AT-2-treated HIV does not alter HCV protein expression

**Figure 5:**
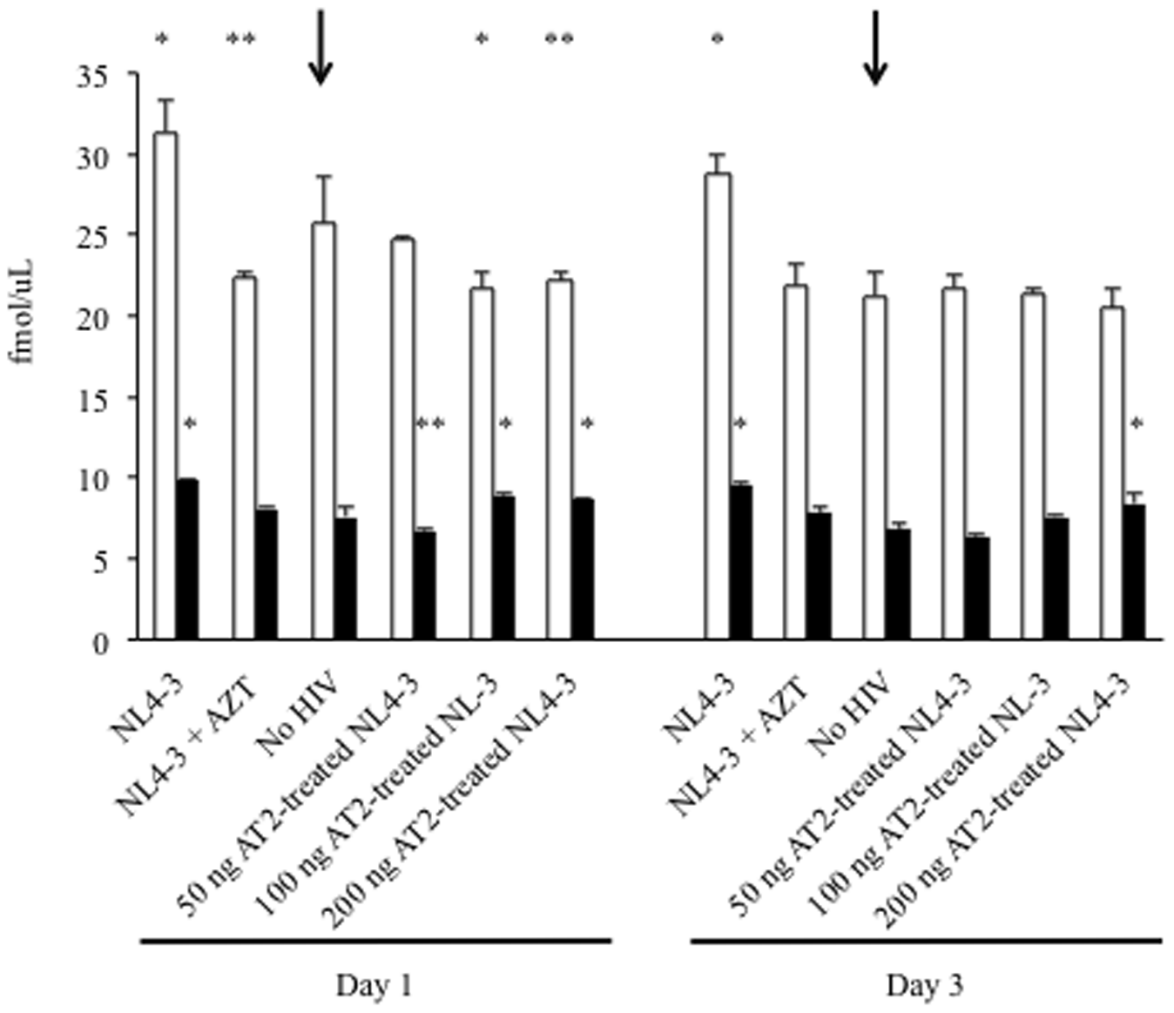
Chemically inactivated HIV does not increase HCV protein expression. 100,000 Huh7.5_JFH1_ cells were incubated with infectious HIV_NL4-3_ alone, infectious HIV_NL4-3_ + 100 uM AZT, no HIV, or AT-2-treated HIV_NL4-3_ (50 ng, 100 ng, or 200 ng) at an MOI = 1 for two hours. At days 1 and 3 post-infection, HCV core antigen was quantified in cell lysates (white bars) and culture supernatants (black bars). * p≤0.05; ** p≤0.10 compared to HIV-uninfected Huh7.5_JFH1_ cells at each time point. Arrows highlight the control – no infectious HIV added – condition.

To examine whether HIV replication was necessary to induce HCV expression, Huh7.5_JFH1_ cells were exposed to 0 ng, 50 ng, 100 ng, or 200 ng of AT2-treated HIV_NL4-3_. As shown in Figure 5, the impact of AT-2-treated HIV_NL4-3_ on cell lysate levels of HCV core protein was not markedly increased from Huh7.5_JFH1_ cells grown in the absence of HIV_NL4-3_. Thus, the effects of HIV on HCV protein expression may require ongoing HIV replication in hepatocytes.

## Discussion

HIV is a critical regulator of viral hepatitis *in vivo*. Emerging data also suggests that HIV can directly impact liver disease. Moreover, several recent reports have convincingly demonstrated productive, low-level HIV infection of hepatocytes [24]–[27], [34]. A number of mechanisms have been postulated to explain the adverse effect of HIV on HCV replication (reviewed in [17], [18]). At the cellular level, HIV-HCV interactions may be mediated via secreted HIV proteins that interact with HCV-infected hepatocytes, and/or direct HIV infection of HCV-infected hepatocytes. For instance, IL-8 attenuates the anti-HCV actions of IFN *in vivo* and *in vitro* [35]–[37], and recombinant, monomeric HIV gp120 protein induces IL-8 production in uninfected hepatocytes [38]. Additionally, STAT1 – a critical element of the Jak-Stat signaling pathway – is antagonized by the HCV core protein, resulting in increased HCV expression [39], [40]. Interestingly, recombinant HIV and HCV envelope proteins cooperatively enhance STAT1-mediated apoptosis in uninfected hepatocytes [41]. Similarly, recombinant HIV gp120 can induce apoptosis in hepatocytes by several distinct pathways that involve Fas ligand, the anti-apoptotic molecule AKT, and caspase expression [42], [43]. Subsequent studies have further demonstrated that gp120 also induces TRAIL-mediated apoptosis [44], as well as enhanced STAT1-mediated apoptosis in uninfected hepatocytes [41]. Recombinant gp120 also increases expression of transforming growth factor b1 (TGFb1) – a key mediator of liver fibrosis – and HCV replication *in vitro* [45]. However, these prior studies were limited in one or more ways as they did not utilize infectious HIV or HIV proteins in their native confirmation, or used hepatocyte-derived cell lines that are not HCV permissive and therefore did not examine the impact on HCV replication. Thus, the impact of infectious HIV on HCV replication has not been evaluated *in vitro*.

In the current study, increased HCV replication in the presence of infectious HIV was demonstrated in several ways. First, infectious HIV resulted in elevated levels of HCV positive- and negative-sense RNA at several time points post-infection. This increased expression of HCV replication occurred in the presence of an R5- or X4-utilizing HIV isolate. Replication of HIV was required for this effect as various antiretroviral medications blocked this pro-HCV effect. HCV protein expression was also elevated in the presence of infectious HIV. While HCV core protein levels were elevated at several time points post-infection with infectious HIV, these differences were not statistically significant. Nonetheless, NS3 and NS5A levels were elevated in the presence of infectious HIV as measured by Western Blot, collectively suggesting that HIV infection results in increased production of several HCV proteins. Moreover, increased HCV replication appeared to require replication-competent HIV, as there was a nominal effect on HCV protein levels when HIV was pretreated with AT-2 to make it replication defective. During the preparation of this manuscript, Zhang *et al.* reported similar findings using Huh7.5 cells that were transduced with CD4 prior to infection with HCV [46]. However, our two studies differ as Zhang *et al*. 1) transduced the Huh7.5 hepatocytes with CD4, rather than relying on Huh7.5 hepatocytes in their “natural” state with a low level of CD4 expression, and 2) utilized Huh7.5 hepatocytes (not constitutively expressing HCV RNA and protein) rather than the Huh7.5_JFH1_ hepatocyte cell line that constitutively expresses HCV RNA and protein). Moreover, we also evaluated HCV protein expression and/or HCV RNA synthesis in the presence of infectious HIV or AT-2 treated virus. Thus, we feel that our model more accurately reflects the hepatic environment *in vivo*.

It is well documented that HCV RNA levels are higher in the presence of HIV co-infection *in vivo* [47]–[49]. While this may be due in part to immune-mediated effects, direct virus-virus interactions are also likely. Thus, HIV suppression may be a critical factor in controlling liver disease, particularly if the underlying liver disease is not treated. Moreover, characterization of the intracellular pathways by which infectious HIV proteins impact liver cell function and/or replication of hepatitis viruses will significantly improve our understanding of HIV pathogenesis and may ultimately improve treatment modalities for HIV-mediated liver disease.
